# Comfort versus Appearance

**Published:** 1916-10-07

**Authors:** 


### The Hospital

The Workers' Newspaper of Administrative Medicine and Institutional Life, Administration, National Insurance and Health.

No. 1583, Vol. LXI. SATURDAY, OCTOBER 7, 1916. PRICE ONE PENNY.

230057

### COMFORT versus APPEARANCE.

Lately a new Chief of the Staff was appointed to direct the operations of the German armies. The same thing has happened before, and on a previous occasion the newly installed chief discovered a sentinel walking to and fro in front of a Government building that was not entitled to have a sentry. At once the German thoroughness, which is perhaps not far distant from fussiness, was in arms, and inquiries were made to find out why the sentinel was there. Books were consulted, archives were searched, pigeon-holes were ransacked, officials were cross-examined, and after prolonged investigation it was discovered that, years before, the railings in front of the building had been repainted, and the Government, in its fussy solicitude for the welfare of its citizens, had placed a sentinel to warn passers-by not to soil their clothes with the wet paint. Days passed, and the paint dried; weeks, months, years passed, and the railings were painted again, and again the paint dried; but still the sentinel marched up and down with fixed bayonet in front of the building, still he was relieved every two hours, and so he would have gone on until the crack of doom if it had not happened that a new broom wanted to score off his predecessor.

The same thing or the same kind of thing happens in every institution, and not the least in hospitals. A rule is made in order that a certain purpose may be attained, but once made the purpose is forgotten, and the rule from being a useful servant becomes a tyrannous master, and is observed merely because it is a rule and without any eye to the purpose for which it was made. This is so in all institutions, and the larger the institution the heavier and stricter becomes the tyranny of rules. It is a good rule that wards should be kept neat and tidy, but the ultimate purpose for which hospitals exist is the welfare of the patient, so that when neatness and tidiness conflict with the welfare of the patient, increase his distress, and retard his recovery, neatness and tidiness should be sacrificed. But this is rarely done. Neatness and tidiness and uniformity of appearance are become a religion in hospitals; they are worshipped with fetish worship, and if they conflict with the welfare of the patient, it is usually the welfare of the patient, not the rule, that is sacrificed.

It is, for instance, a rule that the beds must be kept neat and tidy, and uniform in appearance. The quilts must all hang to the same distance of the floor, the clothes must all be folded down at just the same level on the patient's chest, the surface must be as smooth as if there was no patient occupying the bed. The patient is there, it is true, but as far as his person interferes with the smoothness of the bed-clothes, he is felt to be rather an intruder and a nuisance. The bed would look so much more neat and tidy without him! His shoulders and neck may be chilled, to be sure, but anything is better than an untidy ward, and the ward would look untidy if all the beds were not folded at exactly the same level. The matron would frown and speak to the sister; the sister would upbraid the nurse; there would be a storm in a teacup, the clothes would be folded down, and the patient would have to submit to a chilly neck and shoulders.

When the bed is made, the clothes must be drawn tight across the foot, and firmly tucked in. If the patient is lying on his back, his ankles are over-extended and are held down in this position by the bed-clothes, so that he soon begins to find it painful, and before long very painful. No matter, the bed looks neat and tidy. Not, indeed, as neat and tidy as it would if the bothering patient were not in it; but still, as neat and tidy as it can be made.

The patient is panting and gasping with heart disease, and, with the perpetual restlessness of heart disease, tosses the clothes about and disarranges them. At once a nurse hurries up and puts them straight. No sooner are they put neat and tidy than the unfortunate sufferer tosses them about again. The nurse, in terror lest the sister, or, more horrible, the matron herself, should see an untidy bed, hurries up again and puts the clothes straight. In a few minutes she has the task to do over again, and this time, with the terror of the sister and the matron before her eyes, she cannot help uttering a few words of gentle reproach to the patient. The poor fellow promises to do better, and tries to do better, but things are soon as bad as ever, and now he gets a little relief from his suffer-

WI
H069X

ings by dozing off into a shallow, fitful slumber. The nurse would leave him to sleep if she dared, but there is the sister to consider; there is the matron to dread; there is a black mark waiting for her if either of them sees an untidy bed; so the bed must be straightened and the patient waked, to become once more fully conscious of his misery.

Human nature being what it is, it is perhaps chimerical to hope that this idolatry of rules for their own sake will soon be abolished; nor is it likely that officials brought up under a régime of iron rules will be able to treat them with intelligence; but, nevertheless, it is important for every official who does not wish to subside, as so many officials do, into a mere machine for the observance of rules, to look at rules with intelligence, and to realise that rules are made to achieve a certain purpose; that the unintelligent observance of rules is apt to defeat the purpose for which they were made; that what is important is not the observance of the rule, but the achievement of the purpose; and that no official is competent until he or she can discern when a rule ought to be broken, and, seeing this, has the courage to break it.

What would be the fate of such a nurse as has been sketched if she were to be transferred to private nursing? No private patient would put up with such stupid tyranny, and there is really no reason except a slavish and unintelligent worship of rules why the comfort and welfare of the hospital patient should be sacrificed to appearances.

It must not be supposed that we are advocating disorder and slovenliness in the wards. Neatness and tidiness are excellent things in themselves, and excellent means to the further purpose of prompt and efficient administration; but they are not everything, and they are not paramount. It is the welfare of the patient that is paramount; it is the welfare of the patient for which the rules were constituted and for which they are enforced; and when the enforcement of the rule conflicts with the welfare of the patient, the welfare of the patient must override the rule.

